# Complete Genome Sequences of Nine *Bacillus cereus* Group Phages

**DOI:** 10.1128/genomeA.00473-16

**Published:** 2016-07-14

**Authors:** Samantha Foltz, Allison A. Johnson

**Affiliations:** Center for the Study of Biological Complexity, Virginia Commonwealth University, Richmond, Virginia, USA

## Abstract

We report the sequences of nine novel *Bacillus cereus* group bacteriophages: DIGNKC, Juglone, Nemo, Nigalana, NotTheCreek, Phrodo, SageFayge, Vinny, and Zuko. These bacteriophages are double-stranded DNA-containing *Myoviridae* isolated from soil samples using *B. thuringiensis* subsp. *kurstaki* as the host bacterium.

## GENOME ANNOUNCEMENT

The *Bacillus cereus* group includes 11 closely related species of rod-shaped, sporulating, Gram-positive bacteria (1). The “ATC family” (*B. anthracis*, *B. thuringiensis*, *B. cereus*) are pathogens: *B. anthracis* is an obligate human pathogen, *B. cereus* is an opportunistic pathogen that can cause food poisoning, and *B. thuringiensis* is an insect pathogen used worldwide as a biopesticide. Students at Virginia Commonwealth University, as part of the SEA PHAGES program (2), have discovered, characterized, and annotated the genomes of nine phages infecting *B. thuringiensis* subsp. *kurstaki.* Phages were isolated from soil samples collected across the United States, through the enrichment plating method, and purified by multiple rounds of plaque picking, serial dilution, and plating into *B. thuringiensis* and grown at 30°C.

The reported bacteriophages are *Myoviridae* morphology with head diameters of ~80 nm and contractile tails of ~200 nm in length. The genomic DNA of these bacteriophages was sequenced by 454 or MiSeq next generation sequencing technology to at least 100-fold coverage, assembled by Newbler software, and visualized by Consed. The physical genome ends containing long terminal repeats were determined by identifying a region with approximately double read coverage. DNAMaster (http://cobamide2.bio.pitt.edu/computer.htm) was used to annotate the genes in each *Bacillus* phage genome, and the tool integrates GeneMark (3) and Glimmer (4) for predicting open reading frames and Aragon (5) to predict the presence of tRNA genes. Functional predictions were derived from BLASTp (6) and HHpred (7) and comparing each gene to homologs in Phamerator (8) comparative genomics software.

Sequencing revealed double-stranded DNA genomes of 159,336 to 164,443 bp in length, with G+C content of 37.8 to 38.7%, and long terminal repeat sequences of 2,585 to 3,171 bp in length. Annotation predicted 288 to 302 protein coding genes for each genome. Three of the phages have three to seven tRNA genes.

### Nucleotide sequence accession numbers.

The complete genome sequences of the *Bacillus* phages are available in GenBank at the following accession numbers: DIGNKC, KU737349; Juglone, KU737345; Nemo, KU737352; Nigalana, KU737344; NotTheCreek, KU737351; Phrodo, KU737347; SageFayge, KU737350; Vinny, KU737346; Zuko, KU737348.
